# Toward sustainable outcomes for offshore wind and biodiversity in the digital era: Principles for collaborative digital ecosystem-based governance

**DOI:** 10.1016/j.isci.2026.114881

**Published:** 2026-02-09

**Authors:** Helena Solman, Caitlin Mandeville

**Affiliations:** 1 Environmental Policy Group, Wageningen University, Wageningen, the Netherlands; 2 Gjærevoll Centre, Norwegian University of Science and Technology, Trondheim, Norway

**Keywords:** Environmental science, Environmental management, Environmental monitoring, Energy resources, Ecology, Energy sustainability, energy systems

## Abstract

Digital tools are mushrooming in the renewable energy sector, as a solution for ecological monitoring and as a tool for implementing biodiversity-positive solutions. Despite high expectations for positive impact in sectors such as offshore wind, there has been little consideration of how digitalization shapes biodiversity governance. We argue that realizing the potential of emerging digital technologies for ecological sustainability in the offshore wind sector will require a critical reflection about the assumptions and limitations of digital biodiversity governance approaches. Drawing on literature and examples of digital tools, we argue that the current governance approaches falls short of what’s needed to tackle the ecological impacts of offshore wind. Our perspective proposes four guiding principles for a more collaborative, ecosystem-based way forward. Following these principles could help to realize digital technologies’ promise of sustainable outcomes in the offshore wind sector and in other sectors facing trade-offs between rapid development and ecological impact.

## Graphical abstract

**Figure:**
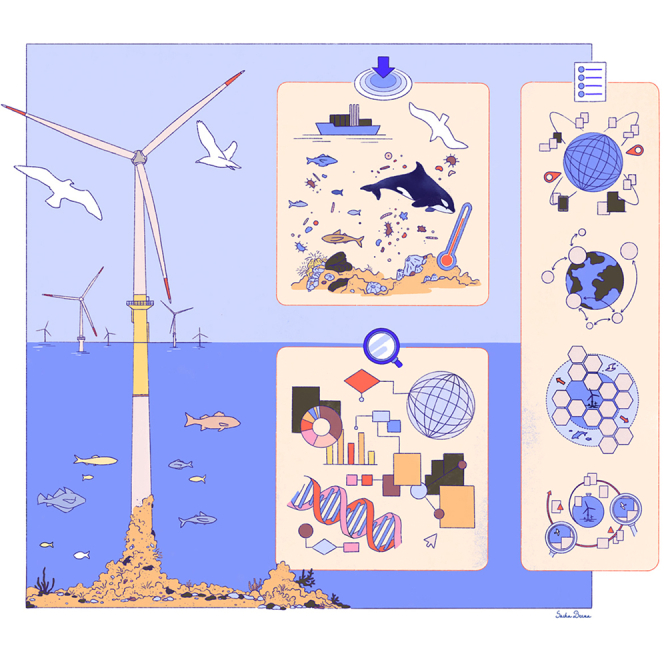

## Introduction

The offshore wind energy sector is a key component of the global renewable energy transition, but growth in this industry has been closely followed by concern about the ecological impacts of wind farm development and operation.^1^^,^^2^^,^^3^ The offshore wind industry has recognized that ecological sustainability is one of the key priorities for research and innovation to reverse the loss of biodiversity,^4^ following the 2022 Kunming-Montreal Global Biodiversity Framework^5^ and European Union Mission on Ocean and Water Restoration.^6^ Ecological concerns also fuel public opposition to offshore wind farms,^7^^,^^8^ making it clear that ecological sustainability is a prerequisite for public support, and hence for fulfilling the ambitions for upscaling of offshore wind farms.

**Figure 1:**
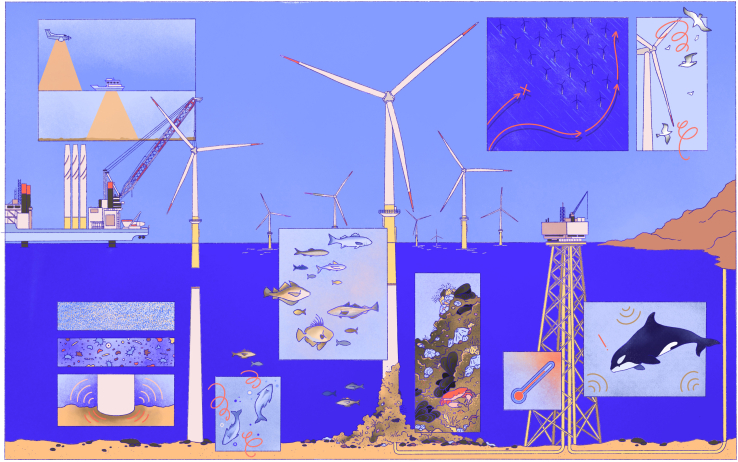
Illustration of direct and indirect impacts associated with offshore wind farm development and operation

Certain ecological effects of wind farm development and operation are increasingly well understood, including negative impacts to many species at the individual- and population-level due to disruptions in ecological processes in the seabed, water column, and air as well as certain positive ecological effects, including artificial reef formation, and reduction of ecosystem pressures due to closure of prior economic activity^3^^,^^9^ (Figure 1). Still, there remains a great deal of uncertainty, especially related to cumulative effects of wind farm operation over long time periods, the interaction of effects from multiple wind farms over a large spatial extent, and the interaction of wind farm impacts with multiple simultaneous stressors.^10^^,^^11^ Progress toward ecological sustainability is hindered by unresolved questions about the nature and magnitude of direct and indirect impacts, how and by whom any impacts should be managed, and, ultimately, whether the climate crisis justifies rapid expansion in the face of ecological uncertainty.^2^^,^^12^

Effective biodiversity governance must engage with these uncertainties (Figure 1). However, strategies for sustaining biodiversity are frequently restricted to a static assessment of potential impacts prior to wind farm construction, often following prescriptions of national conservation laws.^13^^,^^14^ This approach to biodiversity governance leaves little possibility for adaptation in response to emerging environmental impacts. Therefore, marine ecologists have called for measures to improve the capacity to adjust biodiversity governance based on up-to-date information, including monitoring of environmental indicator variables and continuous interpretation of monitoring results.^15^^,^^16^^,^^17^

**Figure 2:**
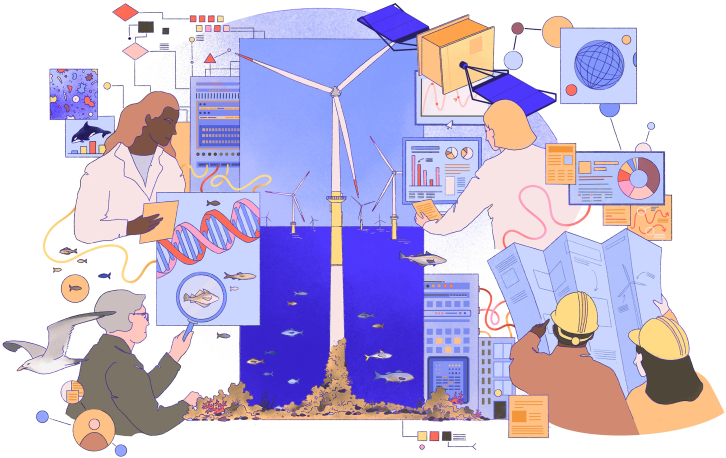
Illustration of proposed features that characterize a collaborative, ecosystem-based approach to digital biodiversity governance in the offshore wind sector

To this end, governments, scientists, and private actors have increasingly developed and applied smart operations and big data gathering and analysis methods.^18^^,^^19^^,^^20^ These technologies include but are not limited to drones, radar, and sensors and cameras on wind turbines, as well as digital infrastructures for managing and interpreting the big data produced by these methods (Table 1; Figure 2). The common expectation is that digital innovation will contribute to better understanding and enhancement of marine biodiversity and thereby foster sustainable coexistence between wind energy development and ecosystems.^21^ But to date, the academic evidence is scattered and there has been a lack of critical evaluation of how digital technologies contribute to solving ecological challenges in offshore wind farms.

**Table 1:** Examples of digital technologies that are emerging in the offshore wind energy sector for the purpose of biodiversity monitoring and management

Digital technology	Application in offshore wind	Examples of projects and companies involved
AI for species recognition	analyzes images and acoustic data to identify and track marine species in wind farm areas	Spoor (software) and Vattenfall (energy company) collaborated to develop AI method for tracking sea birds at Aberdeen Bay Offshore wind farm (Vattenfall Media Relations, 2023)^22^
Animal-borne sensors	records animal movements, characteristics, and/or external environmental variables at the animal’s location in real time via sensors worn by animals	animal-borne sensors tracked migratory movements in species including fishes, birds, and turtles relative to offshore wind farm locations^23^
Environmental DNA (eDNA)	detects the presence of species in water around wind farms to monitor biodiversity and migration patterns, enabled by advanced computational methods	BeWild project, autonomous underwater vehicle that samples eDNA; innovation by Fugro^24^
Radars, sensors, and drones with cameras for smart environments	tracks bird and bat movements to reduce collision risks with turbine blades	the Ecowende project, a joint venture by Shell, Eneco, and Chubu, is deploying these digital technologies to track birds and bats in real-time, to enable shut-down-on-demand to minimize collision risks^25^
IoT-enabled buoys	measures water quality, temperature, and currents to study habitat conditions near turbines	SmartBuoy were developed by Cefas to monitor marine biodiversity changes – the Biodiversity Consultancy used this technology together with a software provided by Spoor at Hywind Tampen offshore wind farm^26^
Autonomous robotics and satellite imaging	maps changes in marine ecosystems, such as seabed habitats and algae growth, around wind farm areas	in collaboration with energy companies, Sensemakers has deployed these technologies to assess the ecological effects of wind farm structures, such as potential reef formations and habitat changes^27^
Digital twins and machine learning	marine imaginary, scenario building, and building of virtual prototypes and “twins” of marine ecosystems for understanding impacts of wind energy and other developments	Azure Digital Twins by SSE Renewables, Microsoft and Avenade develop a virtual environment for monitoring ecological impacts and testing different solutions for mitigation and compensation^28^

To our knowledge, there has been no effort to systematically review such examples; consequently, the table provides examples rather than a comprehensive overview.

Such evaluation has only recently become more feasible with the emergence of research on the general implications of digitalization on biodiversity governance,^29^^,^^30^^,^^31^^,^^32^ and to our knowledge, there have been no attempts yet to zoom into the role of digitalization at the intersection of biodiversity and offshore wind. This is surprising considering that many of the European Union (EU)-level regulatory instruments and funding mechanisms—such as the Digital Europe Program and the European Green Deal—specifically call for inclusion of the social next to the digital aspects in the energy transition and biodiversity is foundational to societal welfare. Meanwhile, offshore wind energy developers increasingly invest in digital tools for biodiversity monitoring and management to develop interventions for net-positive biodiversity impacts.^5^^,^^33^^,^^34^ For example, SSE Renewables, in partnership with Microsoft and Avanade, is launching a digital research initiative off the Dutch coast to study wind farm impacts on the ecosystem, with trials showing promising results in species identification, population monitoring, and distribution analysis.^28^ In contrast, marine ecologists working for non-governmental organisations such as Doggerland in the Netherlands,^35^ emphasize need for conservation without economic developments and warn against possible greenwashing of offshore wind as a “saver” of marine biodiversity.

The result is an emerging paradigm of fragmented digital monitoring with experimental interventions carried out by a heterogeneous variety of public and private actors.^36^^,^^37^^,^^38^ This fragmented research environment is a barrier to iterative learning, adaptation, and upscaling of biodiversity monitoring and management based on direct and indirect impacts of these technologies. Another point of concern is that data from biodiversity monitoring in this fragmented environment are often hard to access and difficult to compare, leaving little capacity to learn about the limitations of what the data represent and how they could be used to inform future strategies for biodiversity.

We argue that realizing the potential of emerging digital technologies for ecological sustainability in the offshore wind industry will require expanding beyond assessment of specific digital technologies and moving toward assessment of the overall shift to digital approaches. This cannot be achieved under the current paradigm of fragmented monitoring and intervention but rather requires a collaborative ecosystem-based approach. To this end, we integrated empirical literature on offshore wind with theory on both digital transitions and adaptive biodiversity management to derive four core principles of a collaborative ecosystem-based approach for digital biodiversity governance of offshore wind. We propose that these core principles can guide more effective adoption of digital technologies in biodiversity governance of the offshore wind industry. Further, we suggest that the process of deriving these principles illustrates how theory on digital transitions can inform sectors facing trade-offs between rapid development and ecological impact more broadly.

## Toward a collaborative, ecosystem-based approach for offshore wind biodiversity governance

The literature on digital environmental governance offers strong theoretical framing to interpret the shift toward digital technologies in the biodiversity governance of offshore wind. Implications of digitalization have now been thoroughly explored in research on other domains of environmental governance; in brief, digitalization tends to transform not only who is engaged in governance but also what is included in the scope of environmental governance and how data are collected, interpreted, and applied.^29^^,^^39^^,^^40^ The work of Kloppenburg et al.^39^ establishes a clear framework to critically evaluate the shift toward digital technologies in biodiversity governance in the offshore wind sector, laying necessary groundwork for assessment and adaptation of these technologies to better realize their intended outcomes.

In this article, we first examine the offshore wind sector in light of the literature on digital transformations to explore underlying causes for the insufficiency of the current digital biodiversity governance paradigm. Next, we integrate theory on digital transitions with literature on adaptive biodiversity management to propose a collaborative ecosystem-based approach that engages more holistically with the direct and indirect impacts of digital technologies on biodiversity governance in the offshore wind industry. We argue that this approach helps to evaluate the digital technologies’ promise of moving toward more sustainable outcomes for biodiversity in offshore wind development.

### Digital technologies transform what is included in the scope of biodiversity governance

The integration of new digital technologies causes shifts in biodiversity governance.^41^ Most immediately, digital technologies offer increased capacity for data collection and analysis. From advanced sensors that collect data as diverse as bioacoustics, fish movement, water quality, and much more^42^; to advanced models that automate the interpretation of these data^43^; to the capacity to remotely curtail or alter wind farm operations in response to real-time detection of biodiversity threats,^44^ digital technologies truly revolutionize how much we know, and can do about, the biodiversity crisis.

However, rapid adoption of new monitoring technologies risks an overemphasis on particular variables due to their compatibility with digital monitoring tools, rather than their ecological relevance. As succinctly put by Turnhout and Purvis^45^: “there is the risk of mistaking what is easily counted for what counts.” This has already been observed in the case of offshore wind, with monitoring technologies tending to focus narrowly on a limited number of species.^46^ Neglect of more meaningful monitoring targets could functionally increase the appearance of compliance with biodiversity governance requirements, but would essentially act as a placebo by neglecting additional variables that could more accurately indicate ecosystem health.^47^

These consequences amplify as digitalization supports transition toward algorithmic forms of governance.^48^^,^^49^ Advanced models can act as a “black box,” where the extent of meaning and uncertainty associated with the original variables become lost, hindering further development.^50^ Without critical consideration of the range of variables and perspectives represented in modeling, algorithmic governance practices such as digital twinning can legitimize and reinforce solutions that do not recognize or account for remaining uncertainties.^51^

Finally, the implications of variable selection are exacerbated by the current paradigm of fragmented, *ad hoc* adoption of digital technologies. While localized selection of monitoring variables enables private actors to detect local-scale biodiversity impacts, it creates challenges for coordinated action at broader scales, including evidence synthesis, collaboration, and integrated action, making it difficult to validate impact claims and outcomes of biodiversity enhancing strategies. At the same time, rapid adoption of novel monitoring and management tools comes with a risk that conventional monitoring approaches will be overlooked or abandoned. There is risk in interrupting existing long-term datasets^52^ and reducing investment in higher cost, smaller data, but valuable research approaches.^53^ This includes ecological experiments that investigate causality of impacts, which is essential for designing future monitoring and interventions.

### Digital technologies transform how biodiversity data are collected, interpreted, and applied

An additional promise of digital biodiversity monitoring is the capacity to extend monitoring over greater spatial extents, supporting research on impacts at broad scales and informing coordinated actions across the boundaries of individual wind farms or government jurisdictions.^54^^,^^55^ Similarly, digital technologies offer the technical capacity for real-time action, automating the process of moving from data to intervention.^44^ However, the literature on digital transitions suggests that the adoption of digital tools is not sufficient to produce these intended outcomes; rather, such adoption must be supported with robust research addressing questions such as Which variables prompt intervention, and how were they chosen? How are thresholds for intervention selected? How will it be determined whether the action was sufficient?

In addition to advancements in data collection, digital technologies also transform the process by which data are analyzed, interpreted, and integrated into governance.^39^ Major examples include an enhanced ability to standardize and share data^56^ and automation of data interpretation and subsequent interventions.^44^ While acknowledging the potential offered by digital approaches, literature on digital transitions highlights a need to research not only the potential benefits of this transformation but also unintended consequences and indirect effects.^41^^,^^57^ For instance, the fragmented nature of offshore wind biodiversity governance can create barriers to data sharing and integration, including insufficient incentivization for private actors.^58^ Ineffective data sharing can lead to redundancy across multiple actors, lack of integrated actions, and even opposing interventions instigated by different actors.^38^ This poses a challenge not only for governments in deciding which interventions are to be prioritized and upscaled, but also for developers who want to avoid the risk of investing in interventions that do not work.

In general, digitalization brings new urgency to questions about who holds power to interpret, shape, and makes decisions about data, and about how intended sustainable outcomes are defined.^39^^,^^59^ Digital data infrastructures allow for the accumulation of huge amounts of data that can become difficult to oversee and understand; as Kloppenburg et al.^39^ state, “data-collection technology has become so powerful that there is a risk of drowning in environmental data.” The sense of control and efficiency conferred by harnessing large amounts of data comes with a risk that decisions about its use in governance are seen as neutral, and that the underlying assumptions and indirect implications are ignored. We argue, however, that in-depth research into these implications will be critical to iterate and improve biodiversity governance of the offshore wind sector.

### Digital technologies transform who participates in biodiversity governance

Lastly, digitalization transforms the range of actors engaged in governance. It is often expected that digital technologies will open governance processes to broader participation. Indeed, digital tools for communication and participation, such as interactive data dashboards, have enabled stakeholders to visualize wind farm impacts and trade-offs in real-time.^40^ However, the potential of digital technologies to democratize governance is coupled with a risk that they can instead reinforce existing power imbalances.^39^^,^^60^ As argued in the strand of literature on responsible research and innovation,^61^^,^^62^ the design and implementation of technologies like digital tools risk reflecting the priorities of technological and scientific experts while marginalizing the perspectives of the stakeholders, such as those of local fishers or conservation advocates. Achieving greater participation requires a critical and collaborative perspective that can identify and respond to these risks.^63^

As has been seen in other sectors,^64^ digitalization has induced shifts from state to non-state or private actors in the biodiversity governance of offshore wind. As private investment in digital technologies has increased, wind energy companies have more often taken on proactive voluntary self-regulation and investment in digital technologies rather than acting mainly in response to government directives.^20^^,^ The independent governance actions of a larger number of private actors have produced a heterogeneous landscape of monitoring initiatives, akin to several small and unstandardized experiments in monitoring and management approaches.^38^ This transition effectively concentrates governance among actors who can afford to invest in participation, while potentially overlooking those without this form of access and limiting the potential for collaborative oversight. This system risks disincentivizing transparency, creating conditions where data and research results may be more likely to be presented if they are seen as demonstrating positive biodiversity impact, while results revealing uncertainties and risks may not be shared. Finally, we caution that a fragmented and uncritical implementation of digital technologies limits the capacity to detect and respond to long-term, cumulative, and indirect effects of wind farms; likewise, a fragmented landscape of digital biodiversity governance is not conducive to evaluating indirect effects of the digitalization process itself. Detection of and response to these impacts requires collaboration that spans outside of the wind industry to include other marine industries, governance entities, conservation researchers, and the public.^9^^,^^11^^,^^12^^,^^65^

## Four principles of collaborative digital ecosystem-based governance

**Figure 3:**
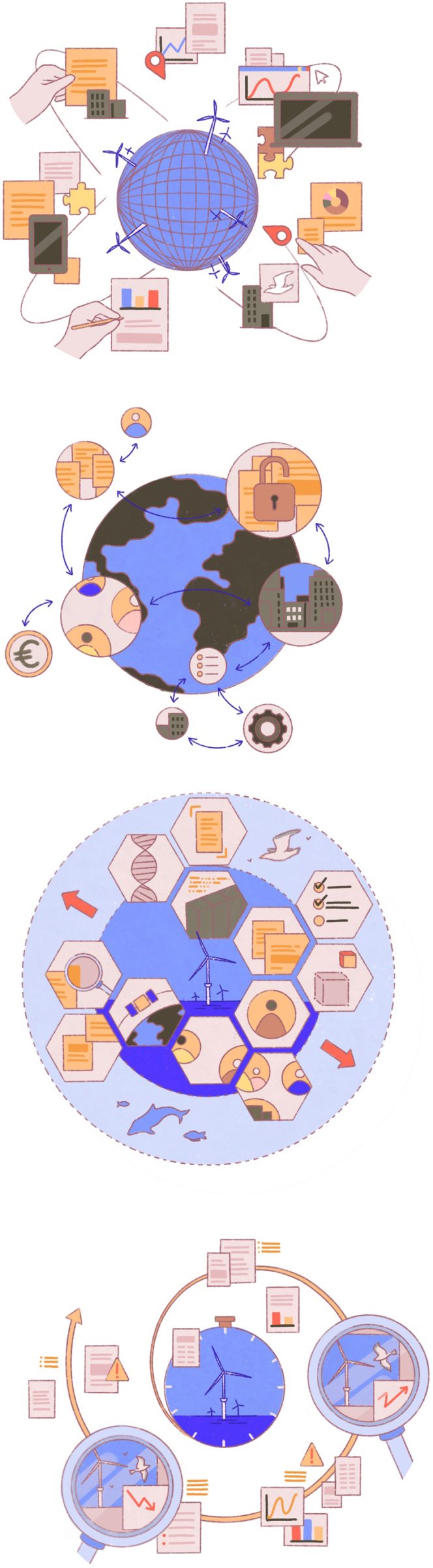
The four proposed principles of collaborative ecosystem-based digital biodiversity governance are (from top to bottom) Setting ecosystem-based biodiversity goals. Standardizing and sharing biodiversity data across borders. Balancing real-time and long-term actions. Inclusive and transparent collaboration.

Based on the abovementioned insights from the literature on digital transitions,^39^^,^^63^^,^^64^ we propose that digital biodiversity governance of offshore wind, along with other sectors of the blue economy, must be implemented and iteratively evaluated in a collaborative, ecosystem-based approach. As argued above, governance of marine biodiversity in the offshore wind energy sector has not yet taken into account transformations related to digitalization, even though programs such as EU Digital Europe Program specially highlight the digital aspect of energy transition and tends to overlook the importance of societal actors, including experts, decision-makers, and stakeholders involved in biodiversity governance. Our approach responds to this gap while building on the principles of open science and FAIR data^66^ and answering calls to better integrate issues of inclusion, representation and justice, in EU Green Deal policy.^67^ To define our approach, we integrate theory on digital transitions with literature on adaptive biodiversity management, in which biodiversity interventions are iteratively adapted in response to information that is learned through ongoing monitoring and management.^15^^,^^16^ To this end, we propose the following four interrelated principles that characterize collaborative digital ecosystem-based governance (subsequently abbreviated as CoDE governance) of biodiversity in the offshore wind sector (as visualised in Figure 3).

### Setting ecosystem-based biodiversity goals

We argue that CoDE governance will prioritize the complex and dynamic relationships between different species and their habitats when setting goals for biodiversity monitoring and management. Because digital technologies change the biodiversity metrics for which it is relatively easy to collect large quantities of monitoring data, it will be critical to test assumptions about metrics chosen to act as proxies for ecological health^45^ and to invest in efforts to overcome taxonomic and other biases.^3^^,^^11^^,^^68^ This can include both traditional monitoring methods and the incorporation and refinement of newer methods, such as eDNA.^69^ With finite resources for monitoring, participatory and multi-stakeholder methods should be followed to set priorities for monitoring.^46^^,^^70^ Among other considerations, prioritization can address knowledge gaps; practical issues, such as the likelihood of detecting a signal given current monitoring capacities; and the need to balance monitoring goals, such as general and targeted monitoring.^46^ Best practices for adaptive monitoring should be followed to retain the value of long-term datasets while adjusting to new information as needed.^52^

Setting biodiversity goals can be done with a help of advanced modeling approaches that are increasingly used to interpret monitoring data and inform management goals and actions. For example, digital twins could help to optimize measures for biodiversity management by evaluating different strategies, such as turbine shutdowns during high-risk periods for bird collisions,^71^ or to assess their ecological benefits before implementation. Even with thoughtful selection of monitoring variables, the advanced modeling approaches used to draw inference from large monitoring datasets should follow best practices to understand uncertainties in the modeling, validate model outputs, and better understand how results can be extrapolated between wind farms, regions, and other varying contexts. Without sufficient communication among researchers and relevant stakeholders about both the meaning and limitation of modeling results, there are risks on the one hand that models will be rejected as untrustworthy and on the other than that they will be unquestioningly accepted without critical evaluation of the parameters included or uncertainties in model outputs.^72^^,^^73^ Advanced analysis approaches, including digital twins, can be planned to feedback toward data collection; models should be used to identify important knowledge gaps that can inform future monitoring. This iterative process will strengthen the capacity for comprehensive simulations and predictions of ecological impacts.^74^

Setting biodiversity goals with the help of digital technologies should happen alongside both long-running conventional monitoring programs and small-data experimental ecology studies.^75^ There are many examples of experimental studies relying on conventional data collection approaches being used to determine the data needs for future monitoring.^76^^,^^77^ In one illustration of the integration of conventional monitoring into government recommendations, the Scottish government issues clear guidance for continuation of long-running conventional monitoring surveys and for their integration with newer digital approaches.^78^^,^^79^

Finally, we argue that social science research that explores the values and priorities of diverse stakeholders and the public is essential to inform the scope of digital biodiversity governance. This includes shaping the definitions of biodiversity and of sustainable outcomes that are used to set goals for action and monitoring.^12^^,^^45^^,^^70^ Beyond this, diverse social science research is further critical to understand the ways that biodiversity data are taken up into varied components of governance ranging from data management to interpretation to application.^80^ Ultimately, in line with Westerlaken,^41^ we argue that social science should be an integral part of projects that design digital tools for biodiversity governance.

### Standardizing and sharing biodiversity data across borders

CoDE governance also prioritizes the natural boundaries of ecosystems, which rarely align precisely with the boundaries of wind farms or governance units. Digital infrastructures that support standardization, sharing, and integration of data, such as the digital twin of the North Sea, can drive research and interventions that span the boundaries of both individual wind farms and government jurisdictions.^39^^,^^81^ The utility of these data infrastructures is contingent, however, on the inclusion of a wide variety of actors and adherence to best practices, including the FAIR data guidelines.^82^^,^^83^ The broader field of biodiversity monitoring has made substantial progress in envisioning a culture of biodiversity monitoring that is networked, integrative, collaborative, and increasingly digital (e.g., Internet of Animals); this is a sound starting point for developing similar networks in the realm of offshore wind biodiversity governance.^58^^,^^84^^,^^85^

In light of the high level of private investment in digital governance approaches, we emphasize the need for research and policy actions that incentivize the sharing of privately held data. We stress that private companies are expected to benefit from openly sharing biodiversity governance data; high standards for data transparency ensure that companies have greater capacity to respond to public demand for sustainability as a prerequisite for offshore wind development.^5^^,^^8^^,^^38^^,^^47^ Third party boundary organizations that act as a bridge between industry, governments, and societal interest groups can play an important role in facilitating progress in this area; examples include the Responsible Offshore Science Alliance (https://www.rosascience.org/) and the Offshore Coalition for Energy and Nature (https://offshore-coalition.eu/).

Importantly, transboundary data standardization and sharing generates capacity for monitoring and research that is integrated across boundaries. We encourage remote monitoring approaches to be deployed to assess critical biodiversity questions that can only be answered at broad spatial scales, including the cumulative impacts of multiple wind farms, the impact of multiple overlapping stressors, and ecological impacts in whole marine ecological systems such as the North Sea basin.^11^ Currently, academic research partnerships offer especially strong examples of successful transboundary research (e.g., NorthWind [https://www.northwindresearch.no/] and WOZEP [https://www.noordzeeloket.nl/en/functions-use/offshore-wind-energy/ecology/offshore-wind-ecological-programme-wozep/]). These and similar research programs can serve as models for transboundary data sharing, research cooperation, and partnership in development of policy guidance.

The ultimate objective of transboundary collaboration should be the development of coordinated biodiversity governance actions to detect and respond to impacts that span across governance jurisdictions.^54^^,^^86^^,^^87^ This includes building capacity to move beyond monitoring for potential impacts and toward active transboundary conservation practices, mitigation of negative impacts and finally compensation for damage, as a last resort. Examples of such actions include collision avoidance, noise reduction, cooling systems, entanglement mitigation, and habitat enhancement^88^ as well as estimation and management of cumulative ecological effects.^89^ With transboundary action, there is a particular need to invest in capacity building for nations with fewer resources and less power to implement monitoring programs and to ensure that a transboundary perspective is not employed to justify actions that disproportionately reduce environmental or economic health of such nations.^12^^,^^90^

### Balancing real-time and long-term action

CoDE governance further recognizes the temporal dynamics of marine ecosystems at multiple scales. One major promise of digitalization in biodiversity governance is a shortened lag time between data collection, interpretation, and intervention. The most prominent example in offshore wind is “smart” curtailment of wind farm operation in response to automated sensing of acute impacts to avian populations.^44^^,^^71^ While promising, it is essential that technical development of these innovations is followed closely by robust impact assessment, including ongoing critical evaluation of the assumptions built into these automated workflows and of limitations in the range of perspectives that informed their development. There are many critical questions about such workflows that cannot be answered on a technical level: for example, how is an acceptable level of impact determined and how is an optimal balance between energy production and avian mortality defined? Advances in the ability to detect and respond to surpassed thresholds lend urgency to the definition of meaningful biodiversity metrics and the identification of societally and scientifically relevant threshold values, a process which requires engagement across sectors and research disciplines.^70^^,^^91^^,^^92^

While the promise of real-time response to biodiversity impacts has been a focus of digitalization to date, we argue that digital innovation also offers critical capacity to address knowledge gaps related to long-term socioecological impacts. A long-range perspective is essential to understand cumulative impacts and indirect impacts that emerge over long time spans, as well as to avoid a shifting baseline effect that can cause short-term monitoring to miss signals of long-term change. Long-term, cumulative, and indirect impacts of offshore wind remain an area of great uncertainty^3^; these impacts are difficult to research in part because they require a scope of data collection, management, and interpretation that in many cases goes beyond the capacity or interests of any single actor.^93^ Digital technologies therefore offer critical capacity to address these long-range monitoring and management objectives, but this potential can only be realized through a collaborative, ecosystem-based approach.

### Inclusive and transparent collaboration

The ecosystem-based governance described above cannot be effectively realized by single actors in isolation; rather, it calls for collaborative action carried out by a network of actors representing diverse perspectives. Therefore, the final principle of CoDE governance is inclusive and transparent collaboration. Care must be taken to ensure that collaboration networks do not reinforce power imbalances and exclude critical perspectives.^12^^,^^60^ Best practices for collaboration in research and governance should be followed: these include inclusion of diverse perspectives and broad consideration of environmental and societal outcomes^72^^,^^94^; adherence to principles of scientific rigor, including open science^95^; and an iterative, adaptive approach that incorporates multiple lines of evidence and accounts for uncertainty.^58^

While the fragmentation caused by the growing predominance of private actors in offshore wind biodiversity governance does pose challenges for collaboration, we stress that industry actors can play a significant role as actors within a collaborative paradigm.^34^^,^^47^^,^^94^ A strong example comes from the FME NTRANS and FME NorthWind projects in Norway, which conducted a multi-month co-creation process to engage industry partners in transdisciplinary, action-oriented dialogue about developing a role of offshore wind industry in Norway.^96^ Interdisciplinary and international academic collaborations further showcase the effective incorporation of a wide range of research perspectives (e.g., BeWild [https://www.wur.nl/en/project/bewild-measuring-biodiversity-at-offshore-wind-farms.htm] and ULTFARMS [https://ultfarms.eu]).

Understanding how digital technologies can deepen public participation in progress toward sustainable outcomes should be a critical focus for future research. Though offshore wind is a subject of great public interest and opinion,^9^^,^^97^ it is relatively removed from day-to-day visibility for much of the public. Already, interactive digital communication tools have made it easier for stakeholders and the public to visualize data and, in some cases, observe decision processes in real time.^40^^,^^98^ For example, a digital twin has informed low-conflict pathways for offshore wind energy plans in Tasmania, highlighting the potential of these tools for knowledge integration and positive stakeholder and community engagement.^99^

Approaches such as participatory mapping and serious games provide novel ways for stakeholders to engage with wind farm impacts by simulating scenarios and evaluating various decisions. Building on this, we encourage more research on virtual environments and tools that allow stakeholders to interact with complex ecosystems.^100^ For such technologies to contribute to fair outcomes, there is a need to develop meaningful participatory processes to ensure that they are equitable, inclusive, and have the capacity to facilitate authentic participation.^39^^,^^60^ Digital tools for participatory science can also empower the public to participate directly in monitoring.^101^ Although there are many examples of participatory monitoring in marine environments, there is room for much more development specifically focused on offshore wind.^102^

## Implementation of CoDE governance principles in the offshore wind energy sector

In this paper, we proposed a set of principles for collaborative, ecosystem-based governance of offshore wind energy sector. Here, we demonstrate how these principles could be applied at different stages of wind energy systems life cycle: from the stage of design, through the stage of planning and management up until the stage of decommissioning. At each stage, there are different opportunities for using digital technologies to improve decisions about how, where and by whom biodiversity is governed in offshore wind farms. We name examples of tools and speculate about how they could help experts across different fields and stakeholders to collaborate and co-produce knowledge about biodiversity and impacts of offshore wind. This list of examples is not meant to be exhaustive but inspirational for the readers looking to identify how they could apply these principles in their daily work.

First, at the stage of design, we see an opportunity in using digital twins for integration of data on the biodiversity and social aspects of marine environments alongside data related to wind farm infrastructure and operation. Moving beyond digitalization of only technical aspects, experts and stakeholders could provide inputs and gain insights about impact in real time and in future scenarios. While this is surely an innovation that would be useful for the sector, we suppose that it will require public funding and oversight to assure access for stakeholders (e.g., fishers, conservationists, and developers). Oversight could come in the form of a working group of stakeholders and experts whose task would be to identify and define limitations and possible bias in the process of design. This is to ensure that digital twins open up, and not close down, opportunities for conservation and to increase the likelihood of positive impact and co-benefits for stakeholders.

Second, at the planning stage, we see a role for digital tools to contribute to better decision-making about how wind farms can co-exist with biodiversity. On a broader scale, digital tools such as open source platforms could be developed to digitalize and manage all wind energy projects in a given basin, e.g., the North Sea. Such a platform should offer a transparent system with open data, including environmental assessments, stakeholder feedback, and regulation, that are accessible to all parties. It should be available in different languages and funded by public institutions at appropriate governance level, such as national or EU. The innovation of a shared platform for a given basin, the North Sea would allow operators, regulators, and non-governmental organizations to view live data on biodiversity indicators, turbine performance, and compliance metrics.

Furthermore, at the level of single, planned offshore wind farms projects, new digital tools could be proposed by bidders in the tendering process, to test new solutions for biodiversity monitoring and decision-making (e.g., AI for species detection and, remote sensing for habitat mapping). This process can be open to innovation but should include standards for data quality, interoperability, and transparency in tender documents. Additionally, tenders should mandate open data sharing where possible so that regulators and stakeholders can verify findings and proposed innovations can be developed and refined in response to the best available knowledge, thereby informing future experimentation.

Third, at the stage of management, we argue that digital tools could help establish smart wind farms that are operated in a way that minimizes negative and maximizes positive biodiversity outcomes, while creating new insights about operations and impacts. In particular, we see a role for smart curtailment to contribute to more bird and bat-friendly operations, as there is increasing evidence about the effectiveness of the different systems for collision prevention. At the same time, we encourage nuanced assessment of the impacts of solutions like smart curtailment; for example, birds or bats that will need to fly around wind farms are still likely to lose parts of their habitat or incur higher energetic cost due to extra flight time. Ongoing monitoring and refinement of interventions such as smart curtailment is therefore an important objective for digital biodiversity monitoring. As with previous digital innovations, we argue that preferably data gathered about biodiversity should be shared and made accessible for the stakeholders and public.

Finally, at the stage of decommissioning, we suggest a non-digital solution: to appoint a steward or organization to critically evaluate the outcomes and impacts of digital biodiversity innovations and provide recommendations to national governments on how to adjust tenders and biodiversity-specific concerns. This could be incentivized and implemented on voluntary basis, and facilitated by national governments.

## Conclusion

The Kunming-Montreal Global Biodiversity Framework calls for transformative change to reverse the loss of biodiversity and advance a vision of “living in harmony with nature.”^103^ As trade-offs become apparent in society’s response to the paired crises of climate change and biodiversity loss, the biodiversity impacts of the renewable energy sector have become an area of increasing focus.^104^^,^^105^ This has been followed by significant investment in digital technologies for biodiversity governance, much of which is directed toward private companies within the renewable energy sector.^18^^,^^34^ The rapid development of the offshore wind sector, followed by immense investment in digital biodiversity governance innovation, exemplifies this trend.^20^

The success of these innovations is a question of great importance; in a departure from its previous iteration, the 2022 Global Biodiversity Framework explicitly recognized the importance of private companies’ actions in achieving its aims.^34^^,^^103^ Despite high expectations placed on new digital technologies for biodiversity governance, however, there has been little coherent effort to assess the impacts of the rapid digitalization of biodiversity governance in the offshore wind sector. Examining the offshore wind industry in light of the literature on digital transitions in environmental governance, we argue that the emerging paradigm of *ad hoc*, fragmented digital monitoring efforts is incompatible with effective biodiversity governance that is grounded in the principles of adaptive management. To integrate digital technologies into biodiversity governance in a way that meets the strong call to action of the Global Biodiversity Framework, we call for a transition to a collaborative ecosystem-based approach to digital biodiversity governance in the wind energy sector.

We have focused on the case of offshore wind, but the principles of collaborative ecosystem-based governance described here can be applied more broadly toward other sectors facing trade-offs between rapid development and ecological impact, both in the blue economy and beyond. We anticipate that this approach, grounded in scholarship on both adaptive biodiversity management and digital transitions, can facilitate real progress toward the win-win for renewable energy development and biodiversity.

### Limitations of the study

As a perspective piece, this study offers a selective view of the rapidly evolving field of digital biodiversity governance in the offshore wind energy sector. Because developments in policy, technology, and industrial practice are advancing quickly and vary across regions, our account cannot capture all current or emerging approaches. We also acknowledge that multiple ways of seeing, knowing, and governing biodiversity coexist across scientific, technical, regulatory, and local knowledge communities. As such, the insights presented here should be understood as one situated perspective among many, rather than a comprehensive or definitive overview.

## Acknowledgments

We thank Sacha Berna for creating the illustrations used in this paper. We also acknowledge the valuable collaboration of all partners in the HoliDoctor project and gratefully recognize the financial support of the 10.13039/501100003246Dutch Research Council (grant no. KICH1.ED02.20.004). C.M. received funding from the European Union under the 10.13039/100018693Horizon Europe Programme, grant agreement no. 101082004 (DiverSea).

## Author contributions

H.S. developed the initial idea for the perspective and designed the overarching conceptual approach; H.S. and C.M. contributed equally to writing the different sections of the manuscript and to revising and editing it throughout submission and revision; C.M. contributed more extensively to the biodiversity science components, while H.S. contributed more substantially to the social science and wind energy literature and insights.

## Declaration of interests

The authors declare that they have no known competing financial interests or personal relationships that could have appeared to influence the work reported in this paper.
